# Reviewer acknowledgement 2013

**DOI:** 10.1186/1687-9856-2014-1

**Published:** 2014-01-30

**Authors:** Scott Rivkees

**Affiliations:** 1 University of Florida, Gainesville, USA

## Abstract

**Contributing reviewers:**

The Editors of *International Journal of Pediatric Endocrinology* would like to thank all our reviewers who have contributed to the journal in Volume 2013 (2013).

## 

Paul Benitez Aguirre

Australia

Carolyn Bondy

United States of America

Raúl Calzada

Mexico

Susan Carlson

United States of America

Aaron Carrel

United States of America

Francesco Chiarelli

Italy

Peter Clayton

United Kingdom

Louise Cominato

Brazil

Cathrine Constantacos

United States of America

Linda DiMeglio

United States of America

Erica Eugster

United States of America

Cheryl Fryar

United States of America

John Fuqua

United States of America

Michael Haller

United States of America

Melissa Ham

United States of America

Adam Hittelman

United States of America

George Jeha

United States of America

Gudmundur Johannsson

Sweden

Paul Kaplowitz

United States of America

Anna Lauber-Biason

Switzerland

Peter Lee

United States of America

Johannes Lemke

Switzerland

Francesco Massart

Italy

Nelly Mauras

United States of America

Mark Molitch

United States of America

John Parks

United States of America

Edward Reiter

United States of America

Ron Rosenfeld

United States of America

Robert Rosenfield

United States of America

Paul Saenger

United States of America

Dorothy Shulman

United States of America

Jill Simmons

United States of America

H. Peter Soyer

Australia

Paul Thornton

United States of America

Amy Wisniewski

United States of America

